# Knowledge, practice of medical waste classifications and related factors of health staff at Ho Chi Minh City District 4 Hospital

**DOI:** 10.1017/ash.2025.145

**Published:** 2025-09-03

**Authors:** To Thanh Tam, Ngô Đồng Khanh

**Affiliations:** 1 Ho Chi Minh city District 4 hospital; 2 Ho Chi Minh City University of Medicine and Pharmacy

**Keywords:** classification, solid medical waste

## Abstract

**Objectives:** In the face of the complicated developments of the COVID-19 epidemic, the increasing number of cases, accompanied by an increase in the number of personal protective equipment has contributed mainly to the increased amount of medical solid waste. Updating knowledge and practicing the correct classifications of solid medical waste according to regulations is an urgent issue to minimize the risk of pandemic spread, health, and the environment, as well as responding to incidents and exposures. To determine the proportion of health workers with correct knowledge and practice in classifying solid medical waste and related factors at District 4 Hospital, Ho Chi Minh City. **Methods:** A cross-sectional study was conducted on 149 health workers at District 4 hospital in 2022. Self- administrated questionnaires including personal data, 50 knowledge questions and practice checklists for solid medical waste classification were used. Determine the relationship using the Χ2 test, PR, and the 95% confidence interval. **Results:** Health staff have knowledge account for 87.25%; general practice 53,69%. Knowledge of color coding non-infectious hazardous waste accounts for less than 50%. Waste bin cleaning 9.4%, exposure reporting procedures 30.87%. The age group >30, the subclinical departments, the information sources from radio, and friends have a higher rate of practice correctly than the other group, p < 0.05. **Conclusions:** Health staff have correct knowledge account for 87.25%; correct practice account for 53.69%. Health facilities need to maintain training on solid waste classification knowledge, focusing on color coding, symbols, handling and responding to incidents of exposure to medical waste and occupational safety. Fully equipped with different means of communication to instruct, supervision classification, collection and transportation of solid waste to take timely remedial measures.

